# Effects of nitrogen addition and root fungal inoculation on the seedling growth and rhizosphere soil microbial community of *Pinus tabulaeformis*

**DOI:** 10.3389/fmicb.2022.1013023

**Published:** 2022-10-19

**Authors:** Lingjie Xu, Xiaoyun Niu, Xia Li, Yanyan Zheng, Hualei Feng, Qiang Fu, Yong Zhou

**Affiliations:** ^1^Country College of Landscape Architecture and Tourism, Hebei Agricultural University, Baoding, China; ^2^School of Life Sciences, Hebei University, Baoding, China

**Keywords:** dark septate endophytes, ectomycorrhizal fungi, growth characteristics, soil nutrients, soil microorganisms

## Abstract

Nitrogen (N) availability is significant in different ecosystems, but the response of forest plant-microbial symbionts to global N deposition remains largely unexplored. In this study, the effects of different N concentration levels on four types of fungi, *Suillus granulatus* (Sg), *Pisolithus tinctorius* (Pt), *Pleotrichocladium opacum* (Po), and *Pseudopyrenochaeta* sp. (Ps), isolated from the roots of *Pinus tabulaeformis* were investigated *in vitro*. Then, the effects of the fungi on the growth performance, nutrient uptake, and rhizosphere soil microbial community structure of *P*. *tabulaeformis* under different N addition conditions (0, 40, and 80 kg hm^−2^ year^−1^) were examined. The biomass and phytohormone contents of the Sg, Pt and Po strains increased with increasing N concentration, while those of the Ps strain first increased and then decreased. All four fungal strains could effectively colonize the plant roots and form a strain-dependent symbiosis with *P*. *tabulaeformis*. Although the effects depended on the fungal species, the growth and root development of inoculated seedlings were higher than those of uninoculated seedlings under N deficiency and normal N supply conditions. However, these positive effects disappeared and even became negative under high N supply conditions. The inoculation of the four fungal strains also showed significant positive effects on the shoot and root nutrient contents of *P*. *tabulaeformis*. Fungal inoculation significantly increased different microbial groups and the total soil microorganisms but decreased the microbial diversity under N deficiency stress. In summary, exogenous symbiotic fungal inoculations could increase the growth performance of *P*. *tabulaeformis* under N deficiency and normal N supply conditions, but the effects were negative under excessive N addition.

## Introduction

In recent years, atmospheric nitrogen (N) deposition has become one of the important phenomena of global climate change. Studies have shown that atmospheric N deposition has adverse effects on terrestrial ecosystems, and these effects are mainly related to soil N enrichment and increases in available N. In addition, N deposition can change the global carbon cycle and plant diversity, which will have a profound impact on the structure and function of terrestrial ecosystems (Schrijver et al., 2011; Phoenix et al., 2012; Liu et al., 2013). As an important part of terrestrial ecosystems, the soil microbial community plays an important role in the whole forest ecosystem. N deposition changes both the N pools and the carbon/nitrogen (C/N) ratio of the substrates where these microorganisms are found, resulting in corresponding changes in the structure and function of the soil microbial community (Compton et al., 2004; Zhou et al., 2020). The effects on specific microbial populations, such as ectomycorrhizal fungi and dark septate endophytes, will determine the forest plant community structure and key ecosystem processes, such as litter decomposition, N fixation and nitrification (Blaško et al., 2013). N utilization by symbiotic fungi of plant roots is an essential aspect of their ecosystem function, and it is important to understand how these changes affect the N forms used by fungi (Zhang et al., 2019). Therefore, it is extremely important to study the interaction between soil fungi and forest plants in response to the rapid increase in N deposition.

Previous studies showed that Pinaceae trees were obligate to ectomycorrhizal fungi, whereas recent research reported that dark septate endophytes were also the main root-associated fungi of pine (Deng et al., 2020; Gehring et al., 2020; Landolt et al., 2020; Chu et al., 2016, 2018, 2021). It is well known that ectomycorrhizal fungi have tremendous effects on plants, such as facilitating water and nutrient uptake in host plants, improving plant growth, aiding in the accumulation of metabolites, and conferring resistance to host plants against pathogens and other abiotic stresses (Smith and Read, 2008; van der Heijden et al., 2015; Sebastiana et al., 2019; Chu et al., 2021). Ectomycorrhizal fungi can not only affect the host plant but also change the soil microenvironment of the host (Talbot et al., 2008; Chu et al., 2021). Previous studies have found that mycorrhizal symbiosis is affected by N addition (Maaroufi, 2019). When the soil N content is limited or low, it can promote mycorrhizal growth (Nehls and Plassard, 2018). However, with the increase in N input, there were differences in the effects on mycorrhizal symbionts, and the degree of difference depended on the amount and time of N deposition in the ecosystem, initial soil N level, and vegetation type (Lilleskov et al., 2002; Maaroufi, 2019). Some studies have shown that the fruiting body yield and biomass of ectomycorrhizal fungi significantly decrease with the increase in N deposition and that long-term N excess can gradually reduce the species richness of ectomycorrhizal fungi, thus changing the community structure composition of ectomycorrhizal fungi (Lilleskov et al., 2002; Hasselquist and Högberg, 2014). At the same time, the soil microbial activity has a close relationship with the plants, soil pH and soil nutrient content, and thus, excessive N will not only directly affect the abundance, diversity and activities of soil microbes but may also indirectly affect plants or mycorrhizal symbionts by changing the mineral nutrient transformation and availability in soil (Leff et al., 2015; Chen et al., 2019). Therefore, we assume that ectomycorrhizal fungal inoculation under N deposition can not only affect the growth and physiological characteristics of host plants but also have an important impact on the soil microbial community in the forest ecosystem where the host is located.

Dark septate endophytes have a wide ecosystem distribution and variable effects on the growth of host plants (Ruotsalainen et al., 2021). Much research has indicated that dark septate endophytes have a similar function to mycorrhizal fungi (Jumpponen, 2011; Surono and Narisawa, 2017; Santos et al., 2021). Dark septate endophytes are a large group of endophytic fungi in plant roots. They mainly colonize the inside of root cells or their intercellular spaces and form dark septate mycelia and microsclerotia (Jumpponen et al., 1998; Mandyam and Jumpponen, 2005). Normally, dark septate endophytes are not limited to specific plant species, and can colonize plant roots in most taxonomic groups in all major biomes of the world, especially in heavy-metal-polluted, drought, alpine, polar and other adverse ecosystems (Barrow, 2003; Mandyam and Jumpponen, 2005; Knapp et al., 2012). The effects of dark septate endophytes on host plants are variable, depending on the combination of host symbiotic plants and fungal species (Newsham, 2011). Studies have shown that dark septate endophytes can significantly promote plant growth (Mayerhofer et al., 2013; Ban et al., 2017; Surono and Narisawa, 2017), nutrient absorption and the stress resistance of host plants (Mandyam et al., 2010; Vergara et al., 2017; Li et al., 2019). Previous studies on the effects of dark septate endophytic inoculation on host stress resistance mostly considered heavy metal pollution (Likar and Regvar, 2013; Berthelot et al., 2016), drought (Santos et al., 2017; Li et al., 2019; He et al., 2022) and pathogen stress (Khastini et al., 2012; Surono and Narisawa, 2018). For example, dark septate endophytes can be used as biocontrol agents for many pathogenic microorganisms and can reduce the adverse effects of plant diseases on plant growth (Terhonen et al., 2016; Surono and Narisawa, 2018). Additionally, dark septate endophytes can enhance photosynthesis, osmotic regulation, and the antioxidant capacity of host plants (Santos et al., 2017; Zhang Q. et al., 2017; Li et al., 2019) to improve the drought resistance of plants. However, the ecological roles of dark septate endophytes with regards to the soil N status are not well known.

*Pinus tabulaeformis* Carr., a coniferous evergreen tree of Pinaceae, is characterized by a well-developed root system, rapid growth, strong ecological adaptability and stress resistance and is a pioneer tree for vegetation restoration in a variety of stress environments (Liu et al., 2019; Zeng et al., 2020). Research has found that there are a variety of ectomycorrhizal fungi in the roots of *P*. *tabulaeformis* (Hibbett and Matheny, 2009; Wang and Guo, 2010). It has a strong dependence on ectomycorrhizal fungi to absorb water and mineral nutrients through mycorrhizae and improve stress resistance (Huang et al., 2008; Zhang and Tang, 2012; Wen et al., 2017; Zhang H. et al., 2017). In addition to ectomycorrhizal fungi, dark septate endophytes also widely colonize the roots of *P*. *tabulaeformis*, but there are relatively few studies on the symbiotic relationship between dark septate endophytes and *P*. *tabulaeformis* at present (Gehring et al., 2020; Chu et al., 2021). The ecological function of dark septate endophytes and whether they affect the growth and stress resistance of mycorrhizal *P*. *tabulaeformis* require further research. Hence, in this study, we conducted two experiments using two ectomycorrhizal fungal strains and two dark septate endophyte strains isolated from *P*. *tabulaeformis* to test (1) the growth performance under different N addition levels in pure cultures and (2) the effects of inoculation with these fungi on the performance of *P*. *tabulaeformis* plants and the soil microbial community in an inoculation experiment under different N supply conditions. Improving the growth of *P. tabulaeformis* will also be a better use of plant root-associated fungal resources, providing a theoretical basis for maintaining the stability of forest ecosystems. We expect that our results will help determine which inoculated fungi could adapt to the change in the N environment and reveal whether they have the potential for improving the stress tolerance and symbiotic performance of plants under N content variation.

## Materials and methods

### Fungal isolates and plant materials

The four fungi applied in these experiments were isolated from the roots of *P*. *tabulaeformis* in the Wuling Mountain Nature Reserve, Hebei Province (117°17′–117°35′E, 40°29′–40°38′N) and were preserved in the Laboratory of Garden Plant Ecology, Hebei Agricultural University, China. These fungi were identified as two ectomycorrhizal fungi, *Suillus granulatus* (Sg) and *Pisolithus tinctorius* (Pt), and two dark septate endophytes, *Pleotrichocladium opacum* (Po) and *Pseudopyrenochaeta* sp. (Ps), by morphological characteristics and phylogenetic analyses of nuclear ribosomal DNA (nrDNA) internal transcribed spacer (ITS) sequences (Supplementary Figures S1,2). All strains were grown on potato dextrose agar (PDA) culture medium for 2 weeks at 27°C in the dark.

Mature seeds of *P*. *tabulaeformis* were also collected from natural populations in the Wuling Mountain Nature Reserve, China, and stored at 4°C. Before the experiment, the seeds were surface-sterilized for 1 h in 0.5% KMnO_4_ solution and then washed 4 times with sterilized distilled water. After that, the seeds were immersed in 40°C sterilized distilled water for 24 h. The seeds were pregerminated on sterile gauze in Petri dishes (9 cm) at 25°C. During the incubation period, the seeds were rinsed with sterilized water twice a day. Seedlings with fully developed cotyledons were transferred to incubation plates containing autoclaved substrate and incubated in a greenhouse at room temperature. The seedlings were watered regularly to avoid water shortage.

## Experiment 1

### Effects of N addition on the fungi *in vitro*

The growth of the four isolates under different N addition conditions was tested in a preliminary experiment in liquid culture. The experiment was performed under sterile conditions, and the basal medium was a modification of modified Melin-Norkrans (MMN) medium (pH 5.5), in which NH_4_NO_3_ was the only N source. The different N concentrations tested were 0 (N free), 0.0053 (low N), 0.053 (medium N) and 0.530 (high N) g l^−1^. Two disks of mycelium (5 mm) were cut from the edge of actively growing 21-day-old colonies of each isolate and inoculated into a 9-cm Petri dish containing 30 ml of MMN solid medium and into a 250-ml Erlenmeyer flask containing 100 ml of MMN liquid medium. Five replicates were performed per strain per N concentration. Then, the Petri dishes were placed in a thermostatic incubator and incubated on a shaker at 150 rpm at 27°C for 20 days in the dark. Upon harvest, the colony diameter on the solid medium was measured; the fungal mycelia in the liquid medium were filtered and washed with sterilized distilled water, and then the fungal mycelia were weighed after drying to a constant weight at 80°C to determine the biomass production. The filtrate was collected for the analysis of the hormone contents.

### Determination of the hormone contents

The analysis of hormone contents (IAA and GA_3_) was performed using high-performance liquid chromatography (HPLC). Briefly, after incubation for 20 days, 20 ml of filtrate of the liquid culture medium was collected and then purified and concentrated to 2.0 ml using a C18 solid-phase extraction column. HPLC was performed using a Thermo U3000 Syncronis C18 column (250 mm × 4.6 mm, particle size 5 μm) with deionized water and acetonitrile (volume ratio of 3:7) as the mobile phase. The flow rate, injection volume, detection wavelength, and column temperature were 1 ml min^−1^, 20 μl, 210 nm and 30°C, respectively.

## Experiment 2

### Pot experiment of *Pinus* *tabulaeformis*

The pot experiment was performed in a greenhouse using a completely randomized design in a 5 × 3 factorial arrangement with fungal inoculation treatment (noninoculated control (CK), Sg, Pt, Po and Ps) and N addition treatment (N free; medium N; high N) as the variables. Each treatment was replicated five times, totaling 75 experimental pots. Seedlings of *P*. *tabulaeformis* were cultured as previously described. Seedlings of uniform size were selected and transplanted into pots (15 cm in diameter and 18 cm in height, 3 seedlings per pot) containing 2 kg of autoclaved (90 min at 121°C) growth substrate, which consisted of a 1:1 (*v*/*v*) mixture of river sand and soil (< 2 mm). With respect to the fungal inoculation treatments, two 5-mm-diameter fungal mycelial discs cut from a 14-day-old PDA culture medium were inoculated within 1 cm of the roots of the *P*. *tabulaeformis* seedlings (Ban et al., 2017; Li et al., 2019). With respect to the noninoculated treatments, two 5-mm discs removed from PDA medium without any fungi were inoculated. All pots were placed in a greenhouse at a mean temperature of 27°C/22°C day/night, a 14 h/10 h photoperiod and 60% mean relative air humidity.

Thirty days after inoculation, the mycorrhizal infection rate was detected by microscopic examination and the staining methods of Phillips and Hayman (1970). After the formation of mycorrhizae was determined, the N addition treatments were conducted. The different N-level treatments were controlled by the addition of modified Hoagland nutrient solution, with NH_4_NO_3_ as the only N source. Three N concentrations (0 kg hm^−2^ year^−1^, N free, 40 kg hm^−2^ year^−1^, medium N; and 80 kg hm^−2^ year^−1^, high N) were applied to the pots. During the experiment, 200 ml of nutrient solution was added once a week per pot. The positions of the pots were randomly rotated each week to minimize location effects. Finally, the plants were harvested after 6 months of treatment.

### Plant biomass and root morphology traits

Prior to harvest, the plant height and ground diameter in each pot were recorded. Plant shoots and roots were subsequently harvested separately. The roots were washed carefully with deionized water and scanned with a desktop scanner (EPSON Perfection V800 Photo, Japan). Several morphological traits of roots (such as total root length, root surface area, root volume and average root diameter) were determined using the WinRHIZO image analysis system (Regent Instrument Inc., Quebec, Canada; Chen et al., 2012). The roots were collected after scanning, and a few root samples were randomly selected to analyze fungal colonization. The remaining roots and fresh shoots were dried at 80°C to constant weight for at least 48 h to calculate the dry weight and water content.

### Fungal colonization rates

Ectomycorrhizal root colonization rates were estimated by microscopic examination of the root tips in six different root sections (3–4 cm) in each of the seedlings within each treatment. The percentage of root tips with distinct ectomycorrhizal structures was calculated by determining the number of mycorrhizal versus nonmycorrhizal root tips in each of the treatments (Brundrett et al., 1996). To evaluate whether the roots were colonized by dark septate endophytes, the fungal structures within the roots were stained with trypan blue (Phillips and Hayman, 1970) and observed under an optical microscope. For each treatment, approximately 30 1-cm segments of fine roots were randomly selected and placed on slides and then observed under a microscope.

### Mycorrhizal growth response

The mycorrhizal growth response (MGR) of *P*. *tabulaeformis* was calculated according to the following equations based on van der Heijden (2002): if M > NM_mean_, then MGR (%) = 100 × (1 – NM_mean_/M), but if M < NM_mean_, then MGR (%) = 100 × (− 1 + M/NM_mean_), where M is the plant total dry weight in the given replicate of the fungal inoculation treatment and NM_mean_ is the mean total dry weight in the corresponding noninoculated treatment. Positive values for MGR indicated that plant growth was promoted by fungi, and negative values indicated that plant growth was suppressed by fungi.

### Determination of plant nutrient contents

Dried powder samples (approximately 0.1 g) of shoots and roots were immersed in H_2_SO_4_ solution and heated for digestion until colorless and transparent. The cooled digestion solution was diluted to 100 ml by adding deionized water. The N concentrations of the plants were analyzed using the Kjeldahl method, the P concentrations were measured by the molybdenum-antimony colorimetric method, and the K concentrations were determined by atomic absorption spectrophotometry (Bao, 2000).

### Soil microbial community composition

The composition of the rhizosphere soil microbial community was determined by phospholipid fatty acid (PLFA) analysis (Buyer and Sasser, 2012). PLFA analysis is widely used as a measurement of soil microbial biomass and community composition. The separation and identification of extracted fatty acids were performed on an Agilent 7,890 gas chromatograph and 5,975 mass spectrometer (Agilent Technologies, Wilmington, DE, United States) with 19-alkyl acid as the internal standard. The abundance of individual fatty acids was determined as the relative nmol per g of dry soil, and standard nomenclature was used. For the statistical analysis of the PLFA data, the concentrations of fatty acids were summed into different biomarker groups and used to estimate their respective biomasses. The gram-positive (G+) bacterial biomass was calculated as the sum of the PLFAs 14:1ω7c, iso14:0, anteiso14:0, iso15:1ω9c, and iso15:1ω6c. The gram-negative (G−) bacterial biomass was calculated as the sum of the PLFAs 14:1ω9c, 14:1ω8c, 14:1ω7c, 14:1ω5c, 15:1ω9c, 15:1ω8c, 15:1ω7c, and 15:1ω6c. The actinomycete biomass was calculated as the sum of the PLFAs 16:0 10-methyl, 17:0 10-methyl, and 18:0 10-methyl. The fungal biomass was calculated as the sum of the PLFAs 18:1ω9c, 18:2ω6,9c, 18:3ω6,9,12c and 16:1ω5c (Rojas et al., 2016). The sum of the PLFA biomarkers detected in each sample was considered to represent the total biomass of the soil microbial community.

The soil microbial community diversity was calculated with the Shannon–Weaver diversity index (*H*) based on the PLFA profiles using the following formula: *H* = –Σ*P_i_*ln*P_i_*, where *P*_i_ refers to the ratio of the concentration of each PLFA to the total PLFA concentration in one soil sample and *n* is the number of PLFAs detected in each soil sample. Each PLFA was considered to be representative of one species (Frostegård et al., 2010; Gui et al., 2017; Wang et al., 2019).

### Statistical analyses

All analyses of variance (ANOVAs) were performed with SPSS software (Version 21.0, SPSS, Chicago, IL, United States). For the first experiment, two-way ANOVA was performed to analyze the effects of the fungal species (E) and N addition treatment (N) on the colony diameter, biomass and hormone content of the four fungi. For the second experiment, two-way ANOVA was performed to examine the effects of fungal inoculation (E), N addition treatment (N), and their interactions on the dry weight, root morphological traits, nutrient contents and soil microbial community structure. The effect of fungal inoculation on the soil microbial community composition consisting of all the PLFA markers was conducted by using principal coordinate analysis (PCoA) based on Bray–Curtis dissimilarity with R software (3.5.2; R Development Core Team 2015). Permutational multivariate analysis of variance (PERMANOVA) was used to test the effects of fungal inoculation and N addition treatment on community dissimilarity (Chen et al., 2019; Jin et al., 2022). All data in each experiment were tested for normality and homogeneity of variance before statistical analyses. The differences between the means among the different treatments were analyzed by Tukey’s HSD *post hoc* tests. The criterion for statistical significance was *p* < 0.05.

## Results

### Experiment 1

#### Effects of N addition on the fungi *in vitro*

**Figure 1:**
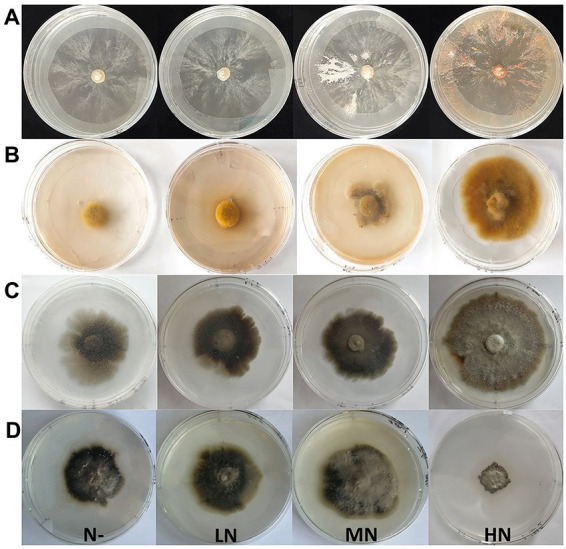
Colony morphology of four fungi under different nitrogen concentrations (N free, low N, medium N, high N). **(A-D)**, Colony morphology of Sg, Pt, Po and Ps under the nitrogen addition treatments. Sg, *Suillus granulatus*; Pt, *Pisolithus tinctorius*; Po, *Pleotrichocladium opacum*; Ps, *Pseudopyrenochaeta* sp.

**Figure 2:**
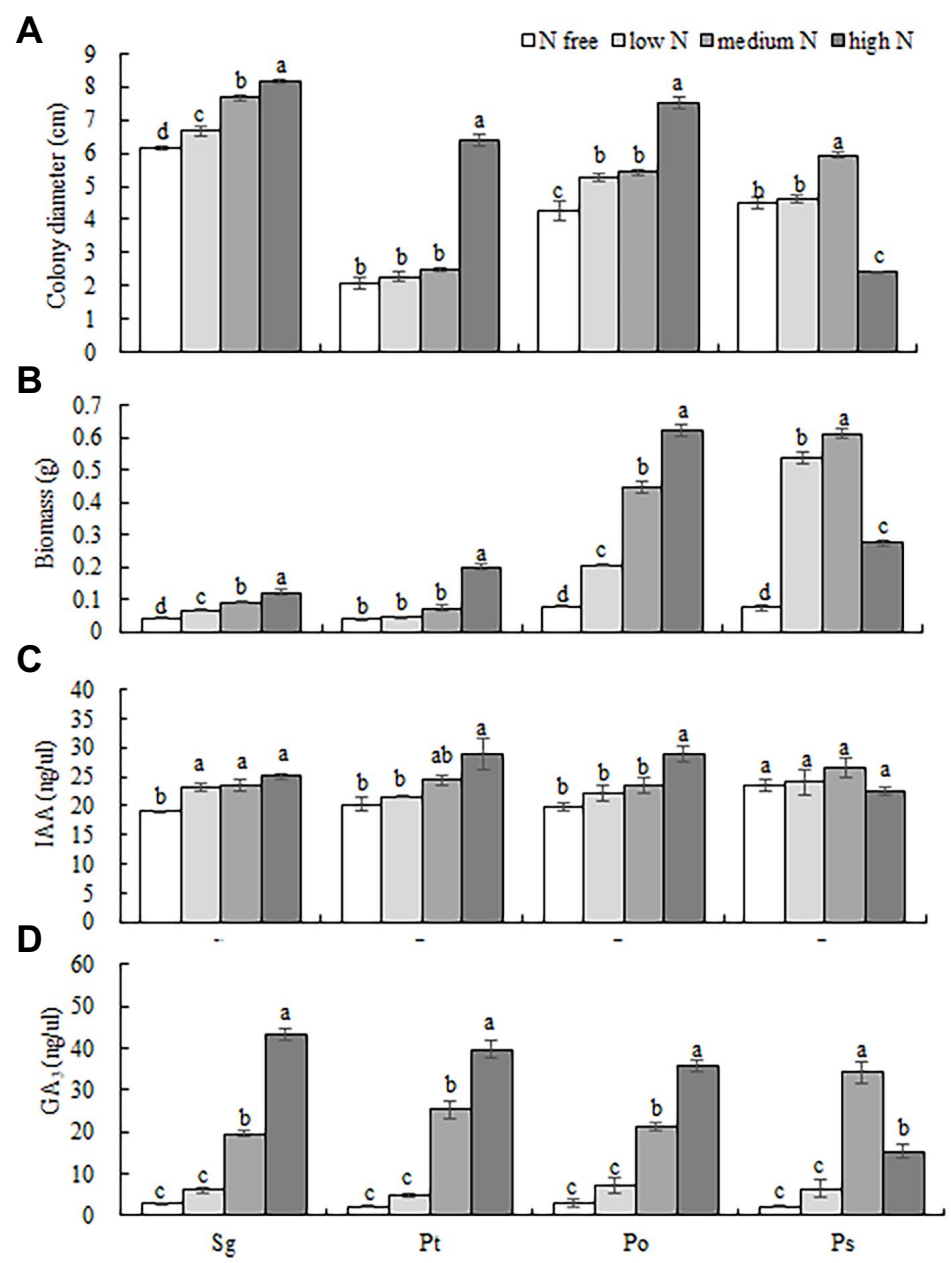
Effects of different nitrogen concentrations (N free, low N, medium N, high N) on the colony diameter **(A)**, biomass **(B)** and hormone contents (IAA and GA_3_) **(C,D)** of four fungi. Different letters above the error bars indicate a significant difference at *p* < 0.05. Sg, *Suillus granulatus*; Pt, *Pisolithus tinctorius*; Po, *Pleotrichocladium opacum*; Ps, *Pseudopyrenochaeta* sp.

Different N concentrations had different effects on the colony diameters of the four fungi (Table 1; Figure 1). The colony diameters of the Sg, Pt and Po strains increased with increasing N concentration; in particular, the colony diameters of the three strains were significantly higher than those of the other treatments under the high N treatment (Figure 2A). The colony diameter of the Ps strain first increased and then decreased with increasing N concentration; the size was the largest under the medium N treatment and significantly smaller under the other N concentrations, particularly under the high N treatment (Figure 2A). The effect trend of the different N concentrations on the biomass of the four fungi was similar to that on the colony diameter. The biomass of the Sg, Pt and Po strains increased with increasing N concentration and was significantly higher under the high N treatment (Figure 2B). The biomass of the Ps strain first increased and then decreased with increasing N concentration, with the biomass of the Ps strain under the medium N treatment being significantly higher than that under the other treatments (Figure 2B). In general, the biomass accumulations of the two dark septate endophytes strains, Po and Ps, were better than those of the two ectomycorrhizal fungi strains, Sg and Pt, under all nitrogen concentrations.

**Table 1:** Analysis of variance (ANOVA) for the effects of fungal species (fungi) and nitrogen addition treatment (N) on the colony diameter, biomass and hormone contents (IAA and GA_3_) of four fungi.

	Fungi		N		Fungi × N	
	*F*	*P*	*F*	*P*	*F*	*P*
Colony diameter	567.317	**<0.001**	136.240	**<0.001**	109.388	**<0.001**
Biomass	388.357	**<0.001**	211.756	**<0.001**	94.645	**<0.001**
IAA	0.962	0.422	14.638	**<0.001**	3.156	**0.008**
GA_3_	2.997	**0.045**	251.392	**<0.001**	18.059	**<0.001**

Significant *p* values are in bold.

The interaction between fungal species and nitrogen concentration significantly affected the contents of the two hormones (Table 1). The contents of IAA and GA_3_ of the Sg, Pt and Po strains increased with increasing nitrogen concentration and reached their highest values under the high N treatment. However, the IAA content of the Ps strain showed no significant difference under different nitrogen concentrations, and the GA_3_ content showed a trend of first increasing and then decreasing with increasing nitrogen concentration, with the GA_3_ content under the medium N treatment being significantly higher than those under the other nitrogen levels (Figures 2C,D).

## Experiment 2

### Growth performance of *Pinus* *tabulaeformis*

Both Sg and Pt could form ectomycorrhizae with *P*. *tabulaeformis* seedlings (Supplementary Figures S3A–F). The front end of the root system of mycorrhizal seedlings was enlarged and thickened and round like a shield. The mycelia of the two dark septate endophytes invaded the cortical intercellular space of the seedling root, grew along the longitudinal axis and formed a loose network structure. Dark septate endophytes also invaded cells and formed a tightly packed cell cluster of enlarged, round, thickened cells inside cortical root cells, forming a microsclerotium structure (Supplementary Figures S3G,H). The noninoculated control did not form ectomycorrhizae or dark septate endophytes structures in the roots of *P*. *tabulaeformis* seedlings. Two-way ANOVA was conducted for each treatment of seedlings, and the results showed that the colonization rate of each treatment reached more than 60%. The seedling colonization rates of Sg, Pt and Po increased with increasing N concentration, while the seedling infection rates of Ps increased first and then decreased with increasing N concentration (Supplementary Figure S4).

**Figure 3:**
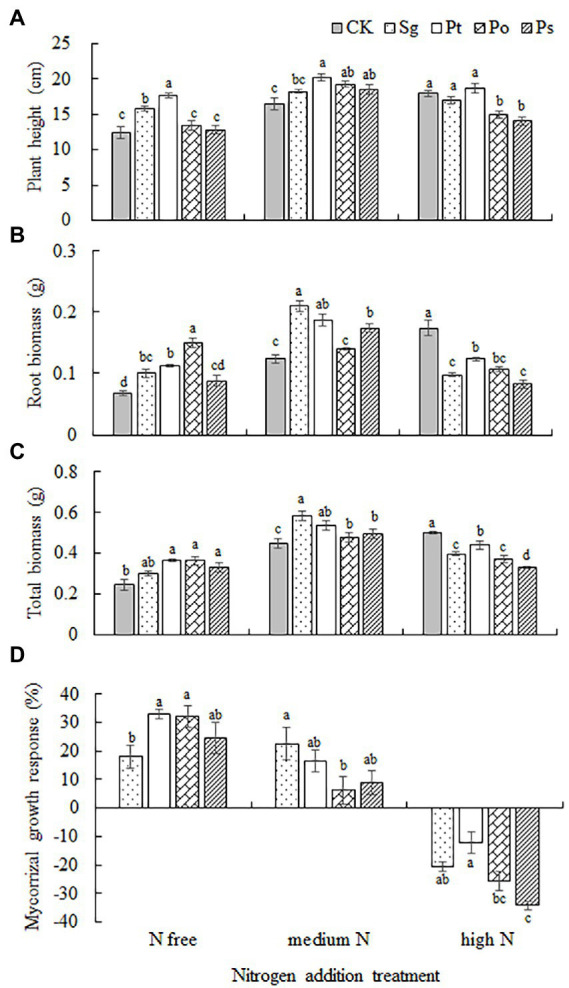
Growth performance of inoculated *Pinus tabulaeformis* under the nitrogen addition treatments (N free, medium N, high N). Different letters above the error bars indicate a significant difference at *p* < 0.05. **(A)**, plant height; **(B),** root biomass; **(C)**, total biomass; and **(D)**, mycorrhizal growth response. Sg, *Suillus granulatus*; Pt, *Pisolithus tinctorius*; Po, *Pleotrichocladium opacum*; Ps, *Pseudopyrenochaeta* sp.

The ground diameter of inoculated seedlings was significantly higher than that of noninoculated seedlings (*p* < 0.05; Table 2). The height, underground biomass and total biomass of seedlings were significantly affected by fungal inoculation, the N addition level and their interaction (Table 2). Under the N-free and medium N treatments, Sg and Pt inoculation significantly increased the plant height and underground and total biomass of *P*. *tabulaeformis* seedlings, while under the high N treatment, both Sg and Pt inoculation decreased the underground and total biomass of the seedlings. Under the N-free treatment, Po inoculation significantly increased the underground and total biomass of *P*. *tabulaeformis* seedlings, while Ps inoculation significantly increased the total biomass. Under the MH treatment, Po and Ps inoculation increased the plant height and underground and total biomass of the seedlings, but Po and Ps inoculation decreased the plant height and underground and total biomass under the high N treatment (Figures 3A–C).

**Table 2:** Analysis of variance (ANOVA) for the effects of fungal inoculation (Fungi) and nitrogen addition treatment (N) on growth and physiological parameters and microbial composition in the rhizosphere soil of *Pinus tabulaeformis*.

	Fungi		N		Fungi × N	
	*F*	*p*	*F*	*p*	*F*	*p*
Fungi colonization rate	45.881	**<0.001**	120.735	**<0.001**	79.895	**<0.001**
Plant height	19.885	**<0.001**	62.675	**<0.001**	6.060	**<0.001**
Ground diameter	8.220	**<0.001**	89.460	**<0.001**	1.975	0.084
Shoot dry weight	1.712	0.173	46.127	**<0.001**	2.140	0.063
Root dry weight	6.979	**<0.001**	115.226	**<0.001**	32.099	**<0.001**
Total dry weight	4.193	**<0.001**	96.866	**<0.001**	8.461	**<0.001**
Mycorrhizal growth response	5.561	**0.005**	179.965	**<0.001**	3.537	**0.012**
Total root length	21.519	**<0.001**	304.060	**<0.001**	4.048	**0.002**
Root surface area	41.821	**<0.001**	98.026	**<0.001**	17.421	**<0.001**
Root volume	27.442	**<0.001**	405.086	**<0.001**	45.573	**<0.001**
Average root diameter	12.492	**<0.001**	35.555	**<0.001**	28.349	**<0.001**
Shoot N concentration	3.311	**0.023**	170.377	**<0.001**	3.087	**0.012**
Shoot P concentration	20.156	**<0.001**	215.700	**<0.001**	3.746	**0.004**
Shoot K concentration	16.939	**<0.001**	251.842	**<0.001**	3.019	**0.013**
Root N concentration	20.005	**<0.001**	93.647	**<0.001**	2.359	**0.042**
Root P concentration	30.805	**<0.001**	210.569	**<0.001**	15.254	**<0.001**
Root K concentration	23.139	**<0.001**	463.239	**<0.001**	34.561	**<0.001**
Gram-positive bacteria	5.266	**0.002**	25.013	**<0.001**	1.919	0.094
Gram-negative bacteria	19.857	**<0.001**	45.345	**<0.001**	0.961	0.484
Fungi	3.807	**0.013**	21.142	**<0.001**	0.406	0.908
Actinomycetes	5.802	**0.001**	86.796	**<0.001**	3.114	**0.011**
Total PLFAs	29.905	**<0.001**	152.590	**<0.001**	2.528	**0.031**
Shannon–Weaver index	1.349	0.275	20.992	**<0.001**	3.203	**0.009**

Significant *p* values are in bold.

There was a significant interaction between fungal inoculation and nitrogen addition treatment on the mycorrhizal growth response of seedlings (Table 2). With the increase in nitrogen concentration, the growth response of Sg seedlings first increased and then decreased, while the growth response of Pt, Po and Ps seedlings decreased (Figure 3D). Under the N-free and medium N treatments, the values of the mycorrhizal growth response were positive, indicating that inoculation had a positive effect on the growth of *P*. *tabulaeformis* seedlings, while the values of seedlings treated by all inoculations were negative under the high N treatment, indicating that inoculation had an inhibitory effect on the growth of seedlings when N was in excess. Under the N-free treatment, the mycorrhizal growth responses of Pt- and Po-inoculated seedlings were significantly higher than that of Sg-inoculated seedlings, while the growth response of Sg-inoculated seedlings was the highest and significantly higher than that of Po-inoculated seedlings under the medium N treatment, indicating that the Sg strain had a better growth effect under the medium N treatment. Under the high N treatment, the value of Pt inoculation was the highest and significantly higher than those of Po and Ps inoculation, while the mycorrhizal growth response of Ps inoculation was the lowest, indicating that Ps inoculation had the highest inhibitory effect on plants (Figure 3D).

### Root morphology and development of *Pinus* *tabulaeformis*

**Figure 4:**
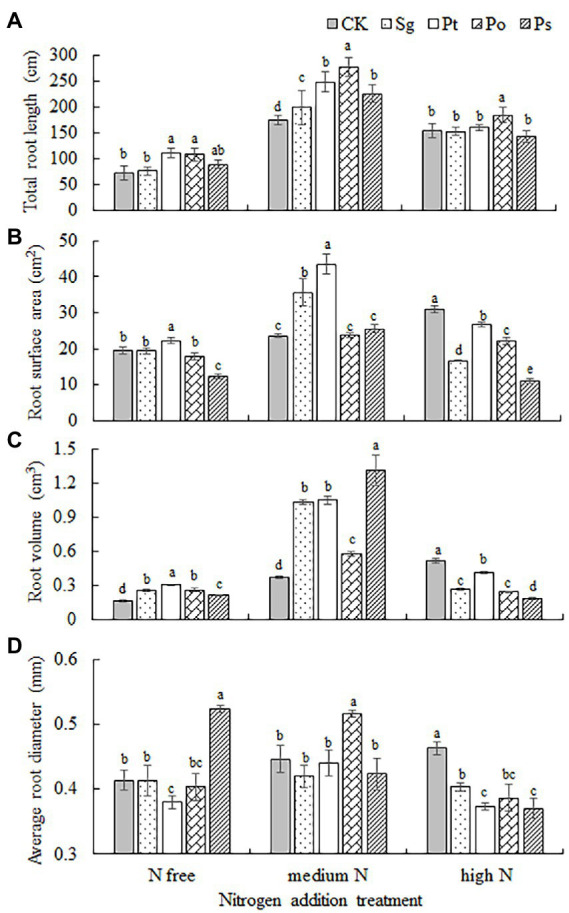
Root morphology of inoculated *Pinus tabulaeformis* under the nitrogen addition treatments (N free, medium N, high N). Different letters above the error bars indicate a significant difference at *p* < 0.05. **(A)**, total root length; **(B)**, root surface area; **(C)**, root volume; and **(D)**, average root diameter. Sg, *Suillus granulatus*; Pt, *Pisolithus tinctorius*; Po, *Pleotrichocladium opacum*; Ps, *Pseudopyrenochaeta* sp.

The total root length, root surface area, root volume and average root diameter of seedlings were significantly affected by fungal inoculation, nitrogen addition and their interaction (Table 2). The root indexes of inoculated seedlings first increased and then decreased with increasing nitrogen concentration (Figure 4). Under the N-free treatment, the root volume of Sg-inoculated seedlings was significantly higher than that under CK; the total root length, root surface area and root volume of Pt-inoculated seedlings were significantly higher than those under CK; the total root length and root volume of Po-inoculated seedlings were significantly higher than those under CK; and the root volume and average root diameter of P-inoculated seedlings were significantly higher than those under CK (Figure 4). Under the medium N treatment, the total root length, root surface area and root volume of Sg- and Pt-inoculated seedlings were significantly higher than those under CK, and the root surface area of plants inoculated with Pt was the largest; the total root length, root volume and average diameter of Po-inoculated seedlings were significantly higher than those under CK, and the total root length and average diameter were also significantly higher than those of the other inoculation treatments; and the total root length and root volume of Ps-inoculated seedlings were significantly higher than those under CK (Figure 4). Under the high N treatment, only the total root length of Po-inoculated seedlings was significantly higher than that under CK, while the root surface area, total volume and root diameter of the other fungus-inoculated seedlings were lower than those under CK (Figure 4).

### Nutrient uptake of *Pinus* *tabulaeformis*

**Figure 5:**
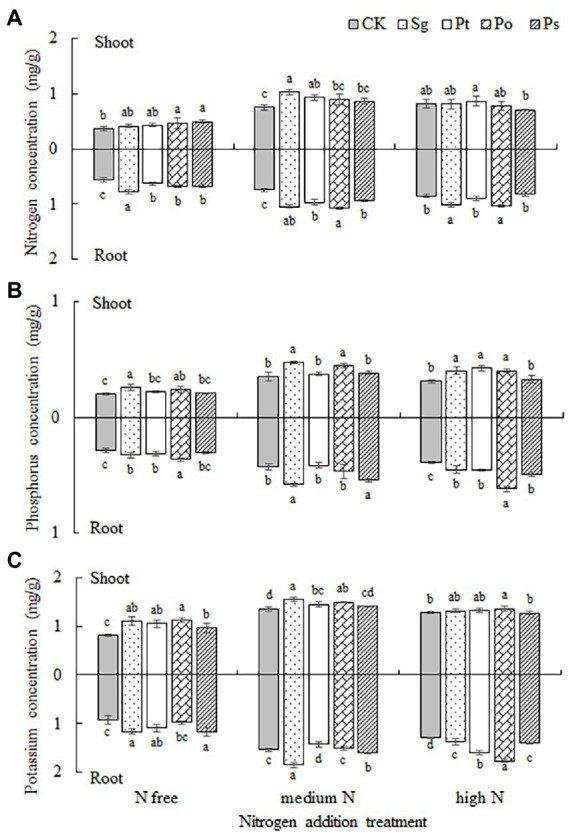
Plant **(A)** nitrogen, **(B)** phosphorus, and **(C)** potassium concentrations in the shoots and roots of inoculated *Pinus tabulaeformis* under the nitrogen addition treatments (N free, medium N, high N). Different letters above the error bars indicate a significant difference at *p* < 0.05. Sg, *Suillus granulatus*; Pt, *Pisolithus tinctorius*; Po, *Pleotrichocladium opacum*; Ps, *Pseudopyrenochaeta* sp.

The effects of fungal inoculation, nitrogen addition treatment and their interaction on the nutrient concentrations in the shoots and roots of *P*. *tabulaeformis* seedlings were significant (Table 2). Under different nitrogen concentration conditions, the effects of inoculation on the nutrient concentrations of host plants were positive in most cases (Figure 5). Under the N-free treatment, the concentrations of N, P and K in Sg-, Pt- and Po-inoculated seedlings were higher than those under CK, and the concentrations of N and K in the shoots and roots of Ps-inoculated seedlings were significantly higher than those under CK (Figure 5). Under the medium N treatment, the N, P and K concentrations were higher than those under CK when the seedlings were inoculated with Sg and Po; the concentrations of N and shoot K in Pt-inoculated seedlings were significantly higher than those under CK, but the concentration of root K was significantly lower than that under CK; and the concentrations of root N, P and K in Ps-inoculated seedlings were significantly higher than those under CK (Figure 5). Under the high N treatment, the plant P and root N and K concentrations of Sg- and Po-inoculated seedlings were significantly higher than those under CK, and the root P and K concentrations of Pt- and Ps-inoculated seedlings were significantly higher than those under CK (Figure 5).

### Soil microbial community composition

**Figure 6:**
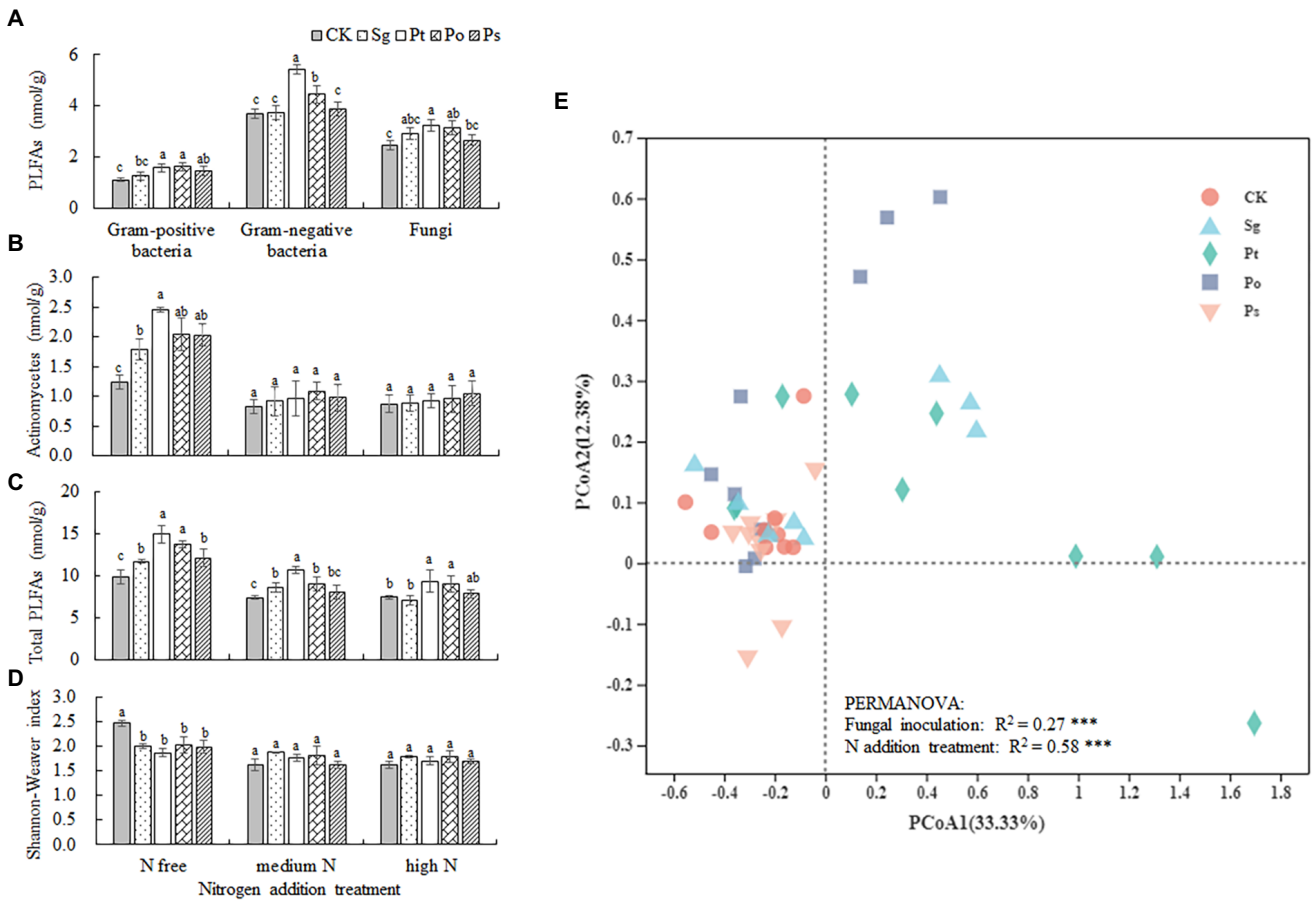
Soil microbial functional group abundance in the rhizosphere soil of inoculated *Pinus tabulaeformis* under the nitrogen addition treatments (N free, medium N, high N). Different letters above the error bars indicate a significant difference at *p* < 0.05. **(A)**, PLFAs of gram-positive bacteria, gram-negative bacteria and fungi; **(B)**, PLFAs of actinomycetes; **(C)**, total PLFAs; and **(D)**, Shannon–Weaver index. **(E)**, Principal coordinate analysis (PCoA) of the soil microbial community. The data consisted of all of the PLFA markers. PERMANOVA statistics refer to significant effects of fungal inoculation and N addition treatment. Sg, *Suillus granulatus*; Pt, *Pisolithus tinctorius*; Po, *Pleotrichocladium opacum*; Ps, *Pseudopyrenochaeta* sp.; ^***^p < 0.001.

There were significant interaction effects between fungal inoculation and N addition treatment on the fatty acid contents of actinomycetes, total PLFAs and Shannon–Wiener index in rhizosphere soil of *P*. *tabulaeformis* seedlings (Table 2). Compared with CK, Pt and Po inoculation significantly increased the contents of G+ and G– bacterial fatty acids and fungal fatty acids in rhizosphere soil, and Ps inoculation significantly increased the content of G+ bacterial fatty acids regardless of N conditions (Figure 6A). Under the N-free treatment, fungal inoculation significantly increased the fatty acid contents of actinomycetes and total PLFAs compared with those under CK, especially when inoculated with Pt, while the Shannon–Wiener index of the soil microbial community was significantly lower than that under CK, indicating that fungal inoculation reduced the microbial diversity in the rhizosphere soil of seedlings (Figures 6B–D). Under the medium N treatment, the total PLFA contents in the rhizosphere soil of Sg-, Pt- and Po-inoculated seedlings were significantly higher than those of CK seedlings, and the same results were also found in the rhizosphere soil of Pt- and Po-inoculated seedlings under the high N treatment (Figure 6C). The results of the principal coordinate analysis (PCoA) showed that the soil microbial community composition of the CK treatment was clearly separated from those of the Pt and Po inoculation treatments (Figure 6E). PERMANOVA showed that fungal inoculation and N addition treatment significantly altered the soil microbial community β-diversity (*p* < 0.001; Figure 6E).

## Discussion

Under adverse conditions, mycelial biomass is one of the important indicators reflecting fungal resistance (Berthelot et al., 2019). Our results showed that the growth of fungi under different N concentrations was related to the fungal species, and the growth of the Sg, Pt and Po strains increased with increasing N concentration. Especially under the high N treatment, the colony diameter and biomass of the three strains were significantly higher than those of the other treatments. The biomass of the Ps strain first increased and then decreased with increasing N concentration. It has long been established that ectomycorrhizal fungi are of critical importance for improving the N nutrition of plants (Read and Perez-Moreno, 2003; Mikusinska et al., 2013). Ectomycorrhizal fungi increase the surface area for absorbing and can act as an extension of the root system (Wu, 2011; Laliberté, 2017), thus storing N nutrition in the mycelium. Dark septate endophytes can also contribute to the capacity of plants to tolerate abiotic stress (Santos et al., 2021). Dark septate endophytes have been isolated from plant roots in many different natural ecosystems, such as arid, temperate, arctic, tropical, boreal, and alpine ecosystems, which are often characterized by abiotic stress conditions (Jumpponen et al., 1998; Mandyam and Jumpponen, 2005; Rodriguez et al., 2009) and have also been found in anthropogenic ecosystems that lack abiotic stress (Andrade-Linares et al., 2011). Several dark septate endophytes were reported to display stress tolerance *in vitro* (Ban et al., 2012; Berthelot et al., 2016; Santos et al., 2017). Therefore, the complex ecological adaptations developed by dark septate endophytes over a long period of evolution contribute to their tolerance to low nutrient conditions. However, in high nitrogen environments, fungi may be limited by other factors, such as carbon (C; Alberton and Kuyper, 2009; Ekblad et al., 2016). The C to N ratio of the substrate has been hypothesized to affect N source absorption by fungi (Maaroufi, 2019). Therefore, in this study, the Ps strain may be limited by carbon in the substrate under the high N treatment, resulting in a decrease in biomass. Studies have found that some rhizosphere microorganisms can produce IAA, GA_3_ and cytokinin substances, which can increase the surface area and number of roots to change the structure of the plant root system. These effects may improve the ability of plants to absorb nutrients from the soil, promote plant growth and development, and resist the negative impact of abiotic stress (Waqas et al., 2012; Priyadharsini and Muthukumar, 2017; Qiang et al., 2019). In this study, the IAA and GA_3_ contents of the two ectomycorrhizal fungal strains (Sg and Pt) and two dark septate endophyte strains (Po and Ps) secreted different concentrations of IAA and GA_3_ under different nitrogen concentrations, indicating that these fungi may have certain growth-promoting potential and produce plant hormones and then release them into the plant tissue.

Fungi can form complex symbiotic relationships with plants and are widely distributed in many ecosystems (Smith and Read, 2008). Studies have shown that a variety of fungi colonize the roots of *P*. *tabulaeformis* and improve the adaptability of host plants to different habitats through complex biological pathways (Huang et al., 2008; Wang and Guo, 2010; Zhang and Tang, 2012; Wen et al., 2017; Zhang H. et al., 2017). However, an understanding of the effects of both fungal colonization and N addition together is lacking. In this study, the four fungi successfully colonized the root tissues of *P*. *tabulaeformis* under all N treatments, and the colonization structures of ectomycorrhizal fungi and dark septate endophytes in the roots were observed, indicating that the changes in N conditions did not affect the effectiveness of colonization of the four fungi. Some previous studies have shown that the mycorrhizal infection rate decreases with increasing soil nitrogen availability (Nilsson et al., 2005; Högberg et al., 2010; Kjøller et al., 2012), while other studies have found that the mycorrhizal infection rate of plants remains unchanged or increases with increasing soil N availability (Wallenda and Kottke, 1998; Kou et al., 2015). Our results suggest that the response of host plants to fungal colonization under different N concentrations was related to the fungal species. The seedling colonization rates of Sg, Pt and Po increased with increasing N concentration, which was consistent with the results of in vitro culture, suggesting that high N availability may be beneficial to the growth and physiological metabolism of mycelia of these three fungi and thus promote their plant root colonization ability. The colonization rate of Ps under the high N treatment was significantly lower than that under the medium N treatment, which was in line with the cost–benefit theory based on reciprocal investments and biological markets (Corkidi et al., 2002; Smith and Read, 2008). With the increase in soil N availability, the dependence of the fungi on plant roots was reduced, and the host plants did not need to obtain more N from the mycorrhiza, resulting in a decrease in the fungal colonization rate.

In this study, inoculation with different fungi significantly promoted seedling height, ground diameter, root length and biomass under the N-free and medium N treatments compared with the CK treatment, while seedling growth indexes were reduced to varying degrees at high N levels compared with CK. The extramatrical mycelium of ectomycorrhizal fungi can increase the surface area for absorbing and could act as an extension of the root system, which is an efficient way to exploit larger volumes of soil beyond the root’s N-depletion zone (Wu, 2011; Laliberté, 2017). Moreover, our results proved that dark septate endophytes can also increase nutritional availability for plants and thus resemble mycorrhizal symbiosis, enabling generally higher growth rates in plants. After plants are inoculated with exogenous fungi, inoculation treatment can help plants cope with the harm of N deficiency to a certain extent. In addition, the mycorrhizal growth response of all inoculation treatments was negative under the high N treatment, indicating that inoculation had an inhibitory effect on the seedling growth of *P*. *tabulaeformis*. Liao et al. (2010) conducted a nitrogen addition experiment on annual Chinese fir, and the results also showed that low nitrogen promoted an increase in seedling biomass, while high nitrogen inhibited seedling growth. Under the condition of adequate soil N availability, the growth-promoting role of inoculated exogenous fungi on *P*. *tabulaeformis* seedlings can be fully played; however, excessive N addition weakens the ability of fungi to infect plant roots, thus affecting the growth of host plants (Wallenstein et al., 2006; Högberg et al., 2010). Therefore, the N input may change the symbiosis between fungi and host plants, as low N and normal N availability would strengthen the symbiosis between fungi and plants to improve the competitive ability of symbionts, while mycorrhizal benefits will be lowest when N, P or other belowground resources do not limit plant growth because plants will tend to reduce C allocation to roots and mycorrhizas in such an environment (Johnson et al., 2010).

The root system is the primary plant part that senses stress conditions, and roots can respond rapidly through changes in elongation (Li et al., 2019; Hou et al., 2020) and function (Shelden et al., 2016; Liese et al., 2018). Several plant growth-promoting microbes, including ectomycorrhizal fungi and dark septate endophytes, have also been shown to influence the root architecture of plants (López-Coria et al., 2016; González-Teuber et al., 2018; Liese et al., 2019; Hou et al., 2020). In this study, we found that the effects of fungal inoculation on the plant root system were dependent on soil N availability. The four fungi promoted the growth of the root system under low and normal N conditions, although the effects depended on the fungal species. Plants inoculated with Pt and Po exhibited a greater root length, and all of the fungi promoted a higher root volume than that of the control plants under the low N treatment. Significantly greater root length, surface area and volume were observed in the plants inoculated with these fungi under the medium N treatment when compared with the control plants, indicating positive effects on root growth. The development of a deep and extensive root system can regulate the absorption of water and nutrients in soil, which ultimately influences biomass production (Hund et al., 2009). This is one of the reasons why ectomycorrhizal fungal or dark septate endophytic inoculation of host plants enhanced the development of the root system under unstressed conditions (Domínguez-Núñez et al., 2006; Wu, 2011; Álvarez-Lafuente et al., 2018; Li et al., 2019; Xiong et al., 2021). In addition, ectomycorrhizal fungi and dark septate endophytes may alter auxin metabolism within the host root, which can regulate root development and change root architecture (Felten et al., 2009; Waqas et al., 2012; Vayssières et al., 2015; Splivallo et al., 2016; Priyadharsini and Muthukumar, 2017).

Nutrient uptake and plant growth are two parameters that are positively correlated. Most studies on ectomycorrhizal fungi-plant interactions revealed positive effects, and some ectomycorrhizal fungi can increase nutritional availability for plants, enabling generally higher growth rates in plants (Dynarski and Houlton, 2020). It has been suggested that dark septate endophytic inoculation also helped host plants absorb more P and N (Jumpponen et al., 1998), as dark septate endophytes can mineralize proteins and peptides in the soil, making N more available for uptake by plant roots (Upson et al., 2009; Vergara et al., 2017, 2018). Our results showed that the effects of fungal inoculation on the nutrient content of *P*. *tabulaeformis* seedlings were dependent on the fungal species and N availability. For all of the fungal inoculation treatments, the effect was almost always positive under the N-free and medium N treatments. A previous study using *P*. *tabulaeformis* found that seedlings inoculated with ectomycorrhizal fungi had a greater biomass and exhibited higher N, P and K contents than nonmycorrhizal seedlings (Lu et al., 2016); Pohjanen et al. (2014) also found that ectomycorrhizal fungi could increase nutrient uptake and growth of Scots pine (*Pinus sylvestris* L.) seedlings. Our study also provided direct evidence that dark septate endophytes may have a similar function to ectomycorrhizal fungi, forming a mutualistic symbiotic relationship with the host (Santos et al., 2021). A large number of extrinsic mycelia in rhizosphere soil can promote the transport, absorption and utilization of mineral elements such as N and P by plants. Dark septate endophytes can promote the growth and biomass accumulation of host plants by decomposing insoluble P and improving the utilization rate of N (Alberton et al., 2010; Vergara et al., 2018; Xu et al., 2020). Hence, the appropriate amount of N addition can increase the soil nutrient utilization of plants in forest ecosystems and reduce the limitation of N deficiency on plant growth.

The rhizosphere microbial community composition is reportedly dependent on the soil nutrient status (Leff et al., 2015; Haas et al., 2018; Chen et al., 2019) and the quantity and quality of root exudates (Marschner and Timonen, 2005; Xie et al., 2019). In this study, the soil microbial community composition could be significantly influenced by the interaction between fungal inoculation and nutrient inputs. The inoculation of the four fungi promoted the abundance of actinomycetes and total soil microorganisms under the N-free treatment, and Pt and Po significantly increased the abundance of soil fungi and G+ and G− bacteria regardless of N conditions. These changes could be related to the modification of growth and nutrient absorption of *P*. *tabulaeformis* after fungal inoculation, as some microbial groups are an important part of the rhizosphere microbial community that can promote plant growth and soil nutrient cycling (Artursson et al., 2006; Carrasco et al., 2010; Sreevidya et al., 2016; Hinojosa et al., 2019). Previous studies have shown that ectomycorrhizal fungi not only help the host plant to improve growth, nutrient conditions and stress tolerance (Smith and Read, 2008) but also recruit and enrich other microorganisms that are beneficial to themselves and/or plants (Cameron et al., 2013; Gupta and Aggarwal, 2018). Ectomycorrhizal fungi or dark septate endophytes may increase the relative abundances of beneficial rhizosphere fungi and bacteria, which are widely reported to promote plant growth through various mechanisms, including biological N fixation, mineral solubilization, iron chelation, and plant growth hormone secretion (He et al., 2019; Chu et al., 2021; Hou et al., 2021). In addition, our results showed that inoculation with exogenous fungi (ectomycorrhizal fungi or dark septate endophytes) reduced soil microbial species diversity under nutrient deficient conditions, which may be due to increased competition between the original fungi and exogenous fungi (Chu et al., 2021).

In summary, our study suggests that the four fungi isolated from *P*. *tabulaeformis* showed good adaptability to different N levels *in vitro*, although the growth performance was dependent on the fungal species. Inoculation with the four fungi improved the growth, root development and nutrient absorption of host plants under the N-free and medium N treatments. However, a high N supply reduced the dependence of host plants on fungi and weakened the symbiotic relationship between fungi and plants. In addition, inoculation with ectomycorrhizal fungi and dark septate endophytes can alter the soil microbial community composition and increase the relative abundances of different microbial groups and total microbial biomass under N deficiency conditions, and these effects might contribute to the improved growth performance of *P*. *tabulaeformis* after fungal inoculation. Therefore, the rational application of N fertilizer and the inoculation of symbiotic fungi play an important role in improving the growth and afforestation of *P*. *tabulaeformis*. Future research needs to address the mechanisms behind the nutrient utilization capacity of root-associated fungi and their involvement in plant growth by a more effective way, such as isotopic leveling method.

## Data availability statement

The original contributions presented in the study are included in the article/Supplementary material, further inquiries can be directed to the corresponding author.

## Author contributions

YZ and XL conceived and designed the experiments and wrote the manuscript, LX, XN, and YYZ performed the research and analyzed the data. HF and QF revised the manuscript. All authors contributed to the article and approved the submitted version.

## Funding

This work was supported by National Natural Science Foundation of China (32001112), Youth Natural Science Foundation of Hebei Province (C2020204169), and the starting Scientific Research Foundation for the introduced talents of Hebei Agricultural University (ZD201728).

## Conflict of interest

The authors declare that the research was conducted in the absence of any commercial or financial relationships that could be construed as a potential conflict of interest.

## Publisher’s note

All claims expressed in this article are solely those of the authors and do not necessarily represent those of their affiliated organizations, or those of the publisher, the editors and the reviewers. Any product that may be evaluated in this article, or claim that may be made by its manufacturer, is not guaranteed or endorsed by the publisher.

## Supplementary material

The Supplementary material for this article can be found online at: https://www.frontiersin.org/articles/10.3389/fmicb.2022.1013023/full#supplementary-material
