# Supplementary material for: Effects of nitrogen addition and root fungal inoculation on the seedling growth and rhizosphere soil microbial community of Pinus tabulaeformis
Source: Front Microbiol. 2022 Oct 19;13:1013023. doi: 10.3389/fmicb.2022.1013023 (PMC9626767; doi:10.3389/fmicb.2022.1013023)
Supplement: Supplementary file 1 [file Data_Sheet_1.pdf]

Supplementary Material

Supplementary Figures

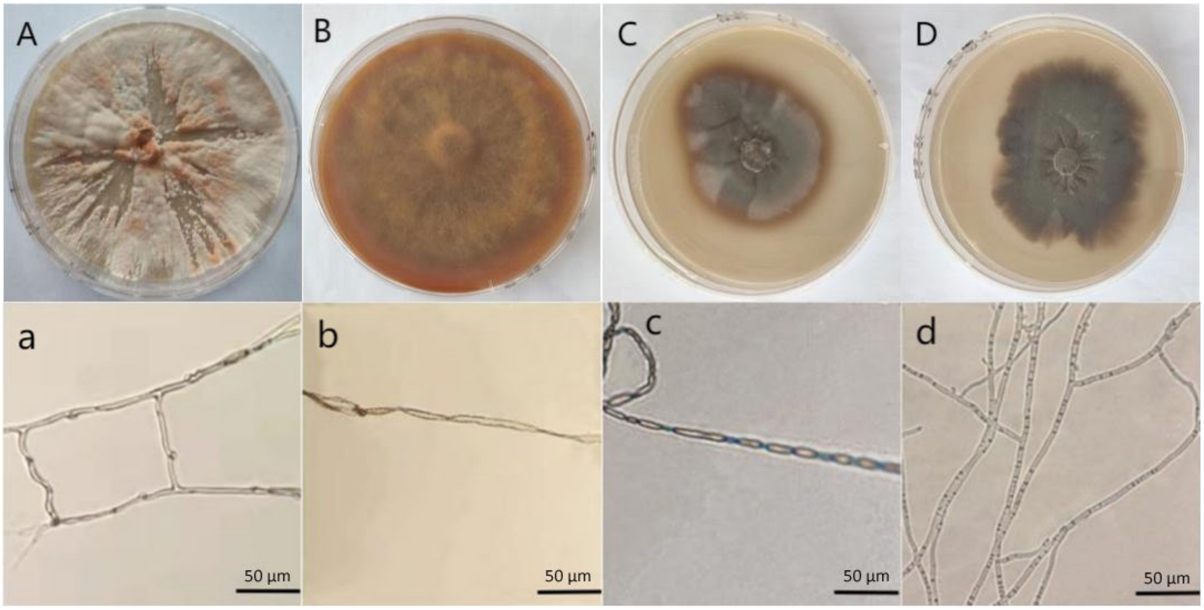

Supplementary Figure S1 Morphology of fungal colonies and mycelium grown on potato dextrose agar (PDA) medium. Note: **A-D**, indicate colony morphology of Sg, Pt, Po and Ps; **a-d**, indicate the corresponding mycelial morphology of Sg, Pt, Po and Ps. Scale bars = 50 µm. Sg, *Suillus granulatus*; Pt, *Pisolithus tinctorius*; Po, *Pleotrichocladium opacum*; Ps, *Pseudoprenochaeta* sp.

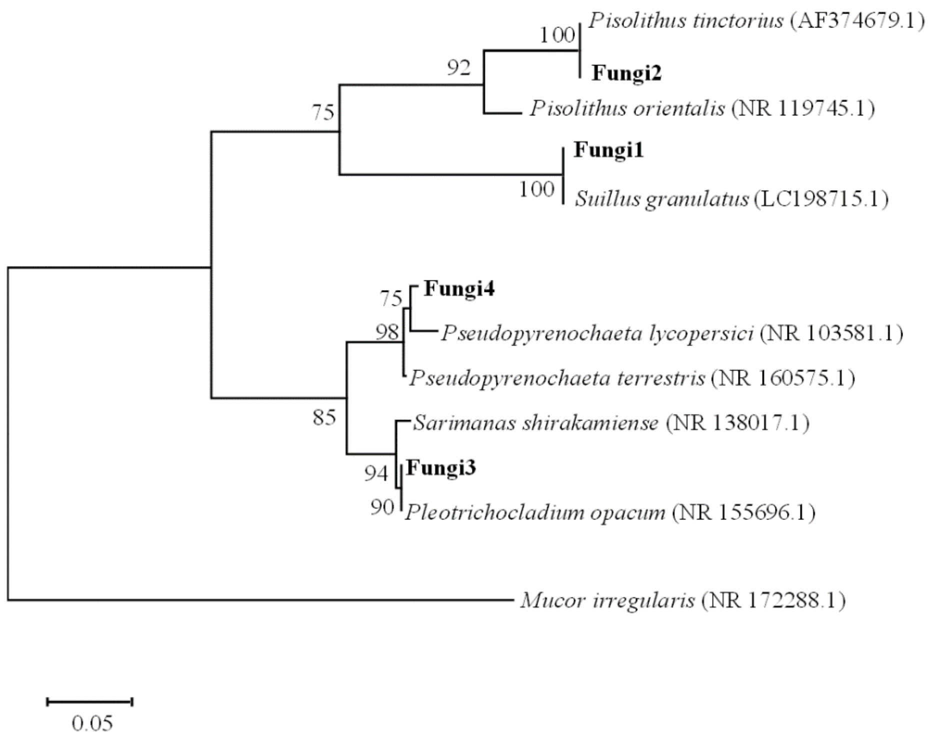

Supplementary Figure S2 Phylogenetic tree based on 18S rDNA gene ITS sequence analysis of fungi isolated from roots of *Pinus tabulaeformis*. Sequences that were determined in the course of this study appear in bold.

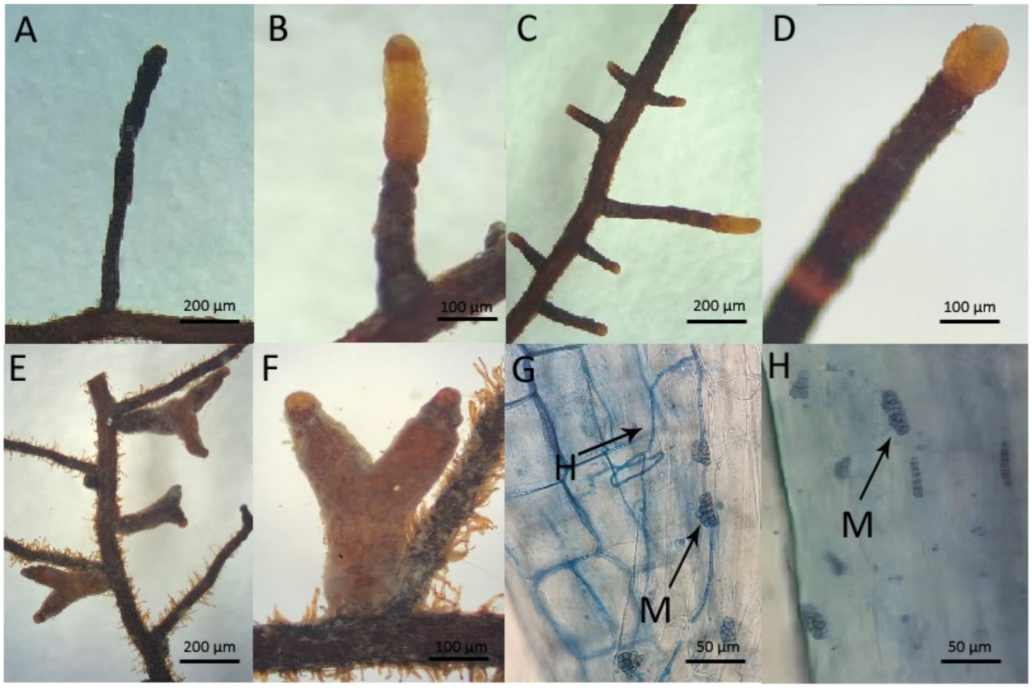

Supplementary Figure S3 Colonization of four fungi in the roots of inoculated *Pinus tabulaeformis*. Note: **(A,B)** mycorrhizae in Sg-inoculated *P. tabulaeformis* roots (Scale bars = 200 and 100 µm), **(C-F)** mycorrhizae in Pt-inoculated *P. tabulaeformis* roots (Scale bars = 200 and 100 µm), **(G)** DSE hyphae and microsclerotia in Po-inoculated *P. tabulaeformis* roots (Scale bars = 50 µm). **(H)** DSE microsclerotia in Ps-inoculated *P. tabulaeformis* roots (Scale bars = 50 µm). H, DSE hyphae, M, DSE microsclerotia. Sg, *Suillus granulatus*; Pt, *Pisolithus tinctorius*; Po, *Pleotrichocladium opacum*; Ps, *Pseudopyrenochaeta* sp.

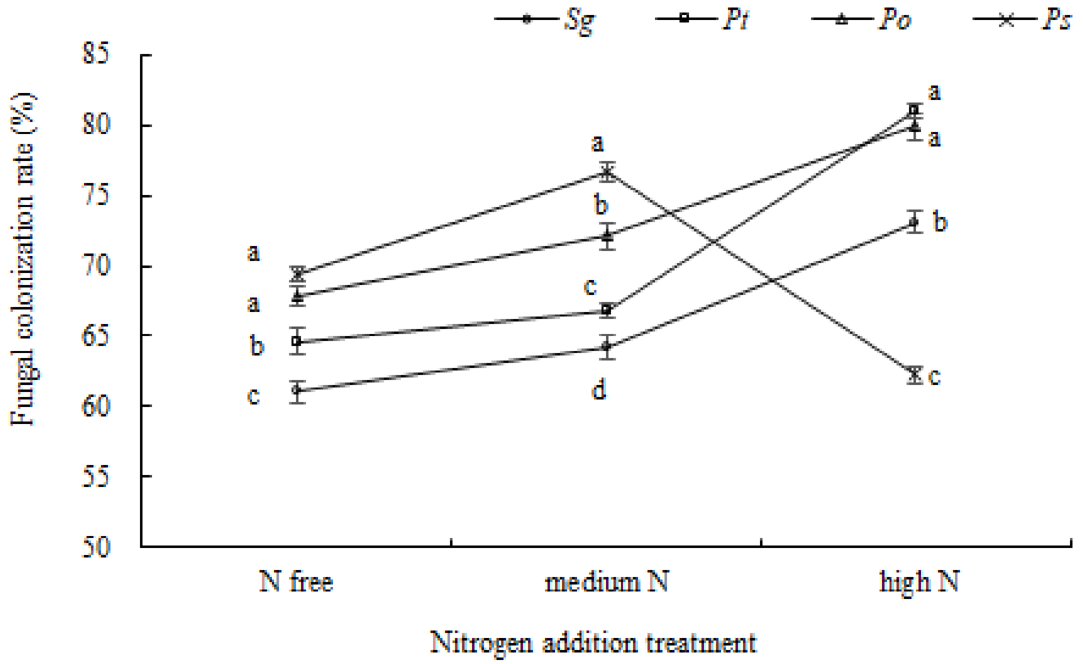

Supplementary Figure S4 Fungal colonization rates of *Pinus tabulaeformis* under different nitrogen concentrations (N free, medium N, high N). Different letters above the error bars indicate a significant difference at $P < 0.05$. Note: Sg, *Suillus granulatus*; Pt, *Pisolithus tinctorius*; Po, *Pleotrichocladium opacum*; Ps, *Pseudopyrenochaeta* sp.
